# The impact of the new ESTRO-ACROP target volume delineation guidelines for postmastectomy radiotherapy after implant-based breast reconstruction on breast complications

**DOI:** 10.3389/fonc.2024.1373434

**Published:** 2024-05-23

**Authors:** Jung Bin Park, Bum-Sup Jang, Ji Hyun Chang, Jin Ho Kim, Chang Heon Choi, Ki Young Hong, Ung Sik Jin, Hak Chang, Yujin Myung, Jae Hoon Jeong, Chan Yeong Heo, In Ah Kim, Kyung Hwan Shin

**Affiliations:** ^1^ Department of Radiation Oncology, Seoul National University Hospital, Seoul, Republic of Korea; ^2^ Department of Radiation Oncology, Seoul National University College of Medicine, Seoul, Republic of Korea; ^3^ Department of Plastic and Reconstructive Surgery, Seoul National University Hospital, Seoul, Republic of Korea; ^4^ Department of Plastic and Reconstructive Surgery, Seoul National University Bundang Hospital, Seongnam, Republic of Korea; ^5^ Department of Radiation Oncology, Seoul National University Bundang Hospital, Seongnam, Republic of Korea; ^6^ Cancer Research Institute, Seoul National University College of Medicine, Seoul, Republic of Korea

**Keywords:** breast cancer, breast implant, breast reconstruction, radiotherapy, target delineation, implant complication, radiotherapy adverse effects

## Abstract

The European Society for Radiotherapy and Oncology–Advisory Committee in Radiation Oncology Practice (ESTRO-ACROP) updated a new target volume delineation guideline for postmastectomy radiotherapy (PMRT) after implant-based reconstruction. This study aimed to evaluate the impact on breast complications with the new guideline compared to the conventional guidelines. In total, 308 patients who underwent PMRT after tissue expander or permanent implant insertion from 2016 to 2021 were included; 184 received PMRT by the new ESTRO-ACROP target delineation (ESTRO-T), and 124 by conventional target delineation (CONV-T). The endpoints were major breast complications (infection, necrosis, dehiscence, capsular contracture, animation deformity, and rupture) requiring re-operation or re-hospitalization and any grade ≥2 breast complications. With a median follow-up of 36.4 months, the cumulative incidence rates of major breast complications at 1, 2, and 3 years were 6.6%, 10.3%, and 12.6% in the ESTRO-T group, and 9.7%, 15.4%, and 16.3% in the CONV-T group; it did not show a significant difference between the groups (p = 0.56). In multivariable analyses, target delineation is not associated with the major complications (sHR = 0.87; p = 0.77). There was no significant difference in any breast complications (3-year incidence, 18.9% vs. 23.3%, respectively; p = 0.56). Symptomatic RT-induced pneumonitis was developed in six (3.2%) and three (2.4%) patients, respectively. One local recurrence occurred in the ESTRO-T group, which was within the ESTRO-target volume. The new ESTRO-ACROP target volume guideline did not demonstrate significant differences in major or any breast complications, although it showed a tendency of reduced complication risks. As the dosimetric benefits of normal organs and comparable oncologic outcomes have been reported, further analyses with long-term follow-up are necessary to evaluate whether it could be connected to better clinical outcomes.

## 1 Introduction

Breast reconstruction has become more widely used to reinstate the breast mound and achieve symmetry with the contralateral breast following mastectomy in patients with breast cancer (1). In addition to a cosmetic issue, it offers long-term psychosocial satisfaction and benefits for quality of life (2, 3). Currently, up to more than 40% of the patients who undergo mastectomy subsequently receive breast reconstruction, with a gradual increase over the past few decades (4). Postmastectomy radiotherapy (PMRT) is widely recommended for patients with locally advanced breast cancer to reduce locoregional recurrence and improve overall survival in those with positive lymph nodes (5–8). However, PMRT may increase the risks of in-breast complications after reconstruction, for example, infection or dehiscence of the wound, deformity or asymmetry of the breast and implant, capsular contracture, and implant rupture, resulting in poor cosmesis and even re-operation (9, 10).

Wide variations in reconstruction surgery and radiation therapy (RT) may make it challenging to facilitate breast reconstruction and postmastectomy RT (PMRT). Both immediate and delayed reconstruction options can be considered for breast reconstruction, and delayed reconstruction could be a one- or two-stage approach depending on the use of a tissue expander before definitive reconstruction surgery. The selection of either autologous tissue or implant is also a major consideration because it can affect reconstruction-related complications (11). When integrating PMRT, there are various factors that may affect reconstruction outcomes, such as RT technique [three-dimensional conformal RT (3D-CRT) vs. intensity-modulated RT (IMRT)], timing (before vs. after reconstruction), or fractionation size (conventional fractionated vs. hypofractionated RT) (12). Among these, hypofractionated RT, which has been increasingly adopted (13) and proved non-inferiority to conventional fractionated RT, also showed comparable breast-related complications after postmastectomy and breast reconstruction (14).

In 2019, The European Society for Radiotherapy and Oncology–Advisory Committee on Radiation Oncology Practice (ESTRO-ACROP) defined a recent target volume delineation guideline for PMRT after immediate implant-based reconstruction endorsed by an international multidisciplinary group of breast cancer experts. While former conventional contouring guidelines generally included synthetic materials such as implant and tissue expander (15), the 2019 ESTRO-ACROP guideline excluded the newly inserted structures from the clinical target volume (CTV) (16). In particular, for patients who received breast reconstruction with subpectoral techniques, CTV consists of only a band-shaped field of cutaneous and subcutaneous tissue located anterior to the implant, which is expected to contain residual breast glandular tissue and lymphatic pathways.

In this context, de-escalating irradiation to the implant capsules and tissues may minimize implant-related complications, such as capsular contracture, animation deformity, and infection. However, there was no clear evidence about the influence of implant-saving RT on in-breast complications in patients who received PMRT after breast reconstruction yet, even though significant tissue-sparing effects for the lung and heart were proved by recent dosimetric analyses (17, 18). This study aims to compare and evaluate breast complications between the delineation by the new ESTRO-ACROP target volume guideline and conventional target volume guideline in patients with breast cancer who received implant-based subpectoral breast reconstruction and hypofractionated PMRT.

## 2 Materials and methods

### 2.1 Patients

After approval by the institutional review boards (IRB No. H-2204–102-1316 and B-2206–760-401), we performed a retrospective chart review of patients with breast cancer who received implant-based reconstruction and PMRT after mastectomy at Seoul National University Hospital and Seoul National University Bundang Hospital between 2016 and 2021. Reconstruction involves temporary tissue expander or direct-to-implant with subpectoral approaches. Our study population excluded individuals who had a prior diagnosis of invasive breast cancer or ductal carcinoma *in situ* on the same side before mastectomy, those who previously received irradiation to the chest wall, those who experienced breast complications that needed operation or hospitalization before PMRT, and those who had not been followed up for at least 6 months. All procedures performed in studies were conducted in accordance with the Declaration of Helsinki on biochemical research involving human participants.

### 2.2 Surgery

All patients underwent reconstructive surgery after mastectomy, with either immediate or delayed-immediate breast reconstruction utilizing subpectoral approaches. Immediate reconstruction was performed concurrently at the time of the mastectomy using permanent prosthetic implants. Delayed-immediate reconstruction, which is a two-stage process, involves the placement of a tissue expander during the mastectomy procedure as the first step. In cases of inflammatory breast cancer, patients underwent delayed-immediate reconstruction. The timing for the final reconstructive surgery was typically between 6 and 12 months after mastectomy and completion of PMRT, which included the use of permanent implants.

### 2.3 Radiation therapy

All patients received PMRT while the tissue expander or implant was in place. PMRT was recommended to be administered within 5 to 6 weeks after mastectomy and reconstruction or 3 to 4 weeks after the completion of adjuvant chemotherapy. If irradiation of a tissue expander was planned, PMRT was administered after the completion of expander inflation. Five radiation oncologists participated in the delineation of target structures. The target structures encompassed the chest wall, three levels of axillary lymph nodes including Rotter’s nodes, and, in cases where necessary, internal mammary nodes (IMN) and the supraclavicular volume (SCV). Regional nodes are delineated according to the previously published guidelines (15).

**Figure 1:**
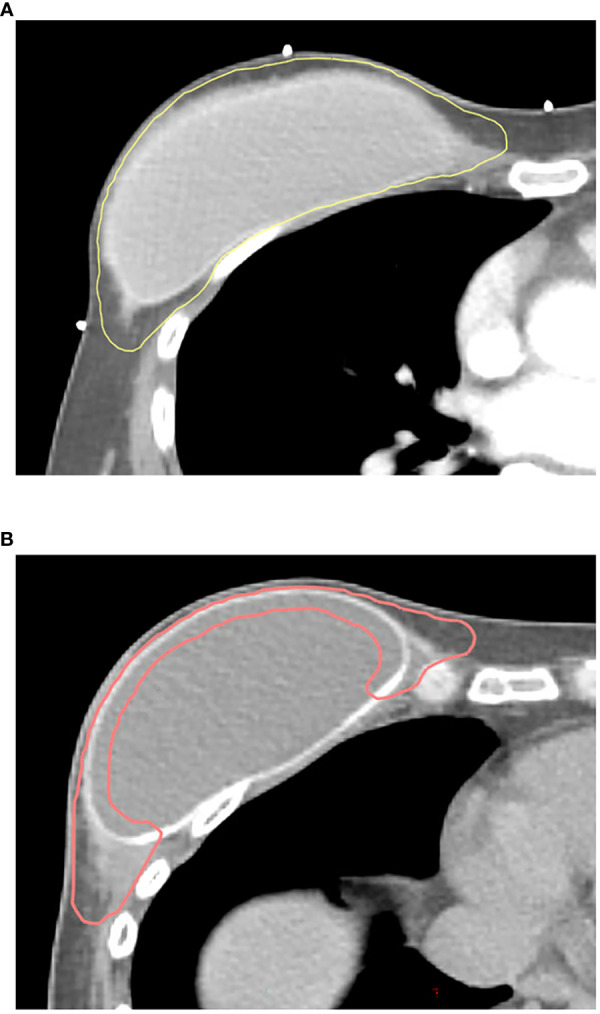
Clinical target volume contours using **(A)** conventional guidelines and **(B)** the 2019 ESTRO-ACROP guidelines. Yellow line, CTV contour with conventional guidelines; pink line, CTV contour with ESTRO-ACROP guidelines.

The chest wall was contoured according to either the new ESTRO-ACROP guideline or conventional guideline for the chest wall. As per the conventional guidelines, the chest wall includes all soft tissue anterior to the pectoral, costal, and intercostal muscles, cropping 3 mm under the surface of the skin with these boundaries. In the new ESTRO-ACROP guideline, the chest wall includes the ventral soft tissue part between the skin and the implant, containing the subcutaneous lymphatic plexus and eventual residual glandular tissue (**Figure 1**). For patients who had a pre-pectoral positioned implant or adverse factors (pT3 disease, non-pathological complete response to systemic therapy, invasion of the major pectoral muscle and/or the chest wall), the dorsal part between the implant and the pectoral muscle/chest wall should be included in the CTV. In our institution, CTV in the ventral region of the implant was delineated with an additional thickness of 3 mm into the implant to cover the entire subcutaneous lymphatic tissues and pectoral muscle over the implant. For the patients who had adverse factors (T4 disease, poor response to chemotherapy, invasion of the pectoral muscle or deeper structures, and inflammatory breast cancer), CTV encompassed the chest wall and the implant like conventional guidelines to include the deep lymphatic channels in the chest wall. Planning target volume (PTV) expansion was 3 mm to 5mm from CTV with avoidance from esophagus and lung: PTV of SCV should not be expanded medially to the esophagus, and the PTV of IMN should not be expanded into the lung and can abut the sternum but not extend into it.

PMRT was delivered with a hypofractionated regimen (2.4–2.7 Gy/fraction), with the dose to the whole reconstructed chest wall of 40.5 Gy to 45.9 Gy, in 15–17 fractions. Regional nodal irradiation was administered at a dose equivalent to the chest wall. When a patient had a lymph node enlargement in the supraclavicular or internal mammary area at the time of diagnosis and did not show a complete response after chemotherapy, a simultaneous integrated boost was done to the post-chemotherapy gross lymph node with 0.5-cm to 0.7-cm margin with a dose of 45.9 Gy to 54.4 Gy. Boost RT was administered on tumors with T4 disease or skin/muscle invasion, sequentially delivered 9.6 Gy to 12.5 Gy in 4 to 5 fractions following irradiation to the chest wall. The target area was expanded 1.5 cm to 2.0 cm around the tumor bed area including the mastectomy scar. Bolus was implemented where the superficial margin was too close or positive to the skin when there was inadequate chest wall tissue in the patient so that a sufficient dose was not prescribed to the chest wall after evaluation of treatment planning.

The 3D-CRT, fixed-field IMRT, and volumetric modulated arc therapy (VMAT) plans were generated using the Eclipse™ (Varian Medical Systems, Palo Alto, CA). Photons generated from a linear accelerator with a range of 6 MV to 10 MV were administered. Two parallel opposed tangential fields were used to treat the chest wall, with an additional anterior field for irradiation of the supraclavicular region in a 3D-CRT treatment plan. In fixed-field IMRT planning, the optimization procedure with the following parameters is detailed in this paragraph. For VMAT planning, multiple continuous beam angles with partial two arcs were utilized depending on the tumor location of each patient. To ensure optimal treatment, at least 95% of the PTV was covered by 95% of the prescribed dose, with the maximum point limited to 105%–107% of the prescribed dose. Dose constraints to the organ at risks (skin, lung, heart, and esophagus) were determined on the basis of the Quantitative Analyses of Normal Tissue Effects in the Clinic, published in 2010, or Radiation Therapy Oncology Group protocols. The ipsilateral lung volume receiving ≥ 20 Gy (V20) was limited to < 20%, and the mean heart dose was mandated to be < 10 Gy. For the contralateral breast, V5Gy was constrained to < 20%.

### 2.4 Definitions of breast complications

The study endpoints consisted of two categories of breast complications: major breast complications and any breast complications. The endpoints were calculated as the time elapsed from the date of the completion of PMRT. Breast complication events included wound-related complications (infection, necrosis, and dehiscence) and implant-related complications (capsular contracture, animation deformity, and rupture). Major breast complications were defined as complications that needed re-operation or re-hospitalization for intervention. More than grade 2 events were categorized as any breast complications. Wound-related complications were defined and graded using Common Terminology Criteria for Adverse Events version 5.0. Capsular contracture was evaluated by the Baker Scale (19). Animation deformity was graded according to the following established criteria: Grade 1, observation; Grade 2, requiring outpatient intervention; and Grade 3, necessitating re-hospitalization or re-operation for intervention. Comprehensive descriptions of all complications were provided and assessed by both a plastic surgeon and a radiation oncologist.

### 2.5 Statistical analyses

We separately analyzed the breast complication rates between ESTRO-ACROP and conventional target groups. We used Student’s t-test and Pearson’s chi-square test to compare patient characteristics between the two target volume delineation methods. The competing risk method was used to evaluate the cumulative incidence of major and any breast complication events. Competing events were defined as re-operation, re-hospitalization, and breast implant removal that were not derived from breast complications. Factors affecting breast complications were evaluated using a Fine–Gray model by univariate and multivariable competing risk regression analyses. In multivariable analyses, target volume delineation and other covariates were included if significant in the univariate analyses at P < 0.20. The RT dose was calculated to an equivalent dose in 2 Gy fractions (EQD2) using an α/β ratio of 3.5. ROC curve was performed to estimate the optimal cut-off values of continuous variables to assess breast complication rates with the greatest predictive capacity. Statistical analyses were performed using R 4.2.1 (The R Foundation for Statistical Computing, Vienna, Austria) with a significance of P < 0.05. The cumulative incidence curves were visualized with STATA software version 17.0 (StataCorp, College Station, TX).

## 3 Results

### 3.1 Patient characteristics

**Figure 2:**
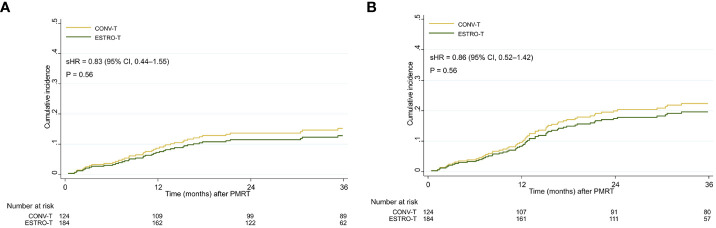
Cumulative incidence curves of **(A)** major breast complications and **(B)** any breast complications in all patients. sHR, subdistribution hazard ratio; 95% CI, 95% confidence interval.

A total of 414 patients with breast cancer underwent curative surgery, of which 308 met the inclusion criteria. Of all the patients, 184 received RT according to the new ESTRO-ACROP target volume delineation (ESTRO-T), and 124 received RT by conventional target volume delineation (CONV-T). Detailed patient, disease, and treatment characteristics of the cohort are presented in **Table 1**. The median follow-up of overall patients was 36.4 months (range, 1.2 months to 83.4 months). In each group, the follow-up duration was 29.4 months (range, 1.2 months to 55.8 months) in the ESTRO-T group and 46.8 months (range, 0.4 months to 83.4 months) in the CONV-T group, with a significant difference (p < 0.001). During the follow-up period, 14 patients underwent breast implant removal for reasons unrelated to breast complications: five for distant metastases, five for dissatisfaction, two for anxiety, and two for unknown causes. Patients in the ESTRO-T group had lower T stages than those in the CONV-T group (p = 0.032). No other differences were found between the two target groups in patient and disease characteristics. For PMRT, the total median dose was 43.2 Gy (range, 42.6 Gy to 60.0 Gy) in the ESTRO-T group and 45.9 Gy (range, 42.6 Gy to 54.4 Gy) in the CONV-T group with a statistically significant difference (p < 0.001). Patients in the ESTRO-T group had a longer interval between preceding reconstruction and PMRT (p = 0.01). IMN irradiation (p < 0.001) and VMAT planning (p < 0.001) were delivered to more patients in the ESTRO-T group. No significant difference was found in the cumulative incidence of all major or any breast complications between the two target volume delineation methods (p = 0.56 and 0.56, respectively) (**Figure 2**).

**Table 1:** Patient characteristics.

	CONV-T (N = 124)	ESTRO-T (N = 184)	Total(N = 308)	P-value
Age (years), mean	45.0 ± 7.9	46.4 ± 8.2	45.8 ± 8.1	0.13
Age (years)				0.13
≥45	63 (50.8%)	76 (41.3%)	139 (45.1%)	
<45	61 (49.2%)	108 (58.7%)	169 (54.9%)	
Follow-up duration (months)	46.8 ± 22.6	29.4 ± 13.4	36.4 ± 19.6	<0.001
Diabetes mellitus				0.73
Yes	3 (2.4%)	7 (3.8%)	10 (3.2%)	
No	121 (97.6%)	177 (96.2%)	298 (96.8%)	
Body mass index (kg/m^2^)				0.77
< 23	71 (57.3%)	101 (54.9%)	172 (55.8%)	
≥ 23	53 (42.7%)	83 (45.1%)	136 (44.2%)	
Smoking history				0.93
Yes	3 (2.4%)	6 (3.3%)	9 (2.9%)	
No	121 (97.6%)	178 (96.7%)	299 (97.1%)	
Laterality				0.11
Left	77 (62.1%)	96 (52.2%)	173 (56.2%)	
Right	47 (37.9%)	88 (47.8%)	135 (43.8%)	
Histologic type				0.54
Intraductal carcinoma	103 (83.1%)	160 (87.0%)	263 (85.4%)	
Intralobular carcinoma	14 (11.3%)	14 (7.6%)	28 (9.1%)	
Others	7 (5.6%)	10 (5.4%)	17 (5.5%)	
T stage (AJCC 8th) ^a^				0.03
T1	10 (8.1%)	24 (13.0%)	34 (11.0%)	
T2	63 (50.8%)	113 (61.4%)	176 (57.1%)	
T3	37 (29.8%)	35 (19.0%)	72 (23.4%)	
T4	14 (11.3%)	12 (6.5%)	26 (8.4%)	
N stage (AJCC 8th) ^a^				0.33
N0	15 (12.1%)	12 (6.5%)	27 (8.8%)	
N1	57 (46.0%)	95 (51.6%)	152 (49.4%)	
N2	37 (29.8%)	51 (27.7%)	88 (28.6%)	
N3	15 (12.1%)	26 (14.1%)	41 (13.3%)	
Molecular type				0.41
Luminal A	70 (56.5%)	101 (54.9%)	171 (55.5%)	
Luminal B1	6 (4.8%)	19 (10.3%)	25 (8.1%)	
Luminal B2	15 (12.1%)	24 (13.0%)	39 (12.7%)	
HER-2 enriched	15 (12.1%)	21 (11.4%)	36 (11.7%)	
Triple-negative breast cancer	18 (14.5%)	19 (10.3%)	37 (12.0%)	
Skin invasion				0.47
Yes	14 (11.3%)	15 (8.2%)	29 (9.4%)	
No	110 (88.7%)	169 (91.8%)	279 (90.6%)	
Nipple invasion				0.88
Yes	15 (12.1%)	20 (10.9%)	35 (11.4%)	
No	109 (87.9%)	164 (89.1%)	273 (88.6%)	
Muscle invasion				0.08
Yes	5 (4.0%)	1 (0.5%)	6 (1.9%)	
No	119 (96.0%)	183 (99.5%)	302 (98.1%)	
Mastectomy				0.07
Nipple-sparing mastectomy	24 (19.4%)	39 (21.2%)	63 (20.5%)	
Skin-sparing mastectomy	86 (69.4%)	137 (74.5%)	223 (72.4%)	
Total mastectomy	14 (11.3%)	8 (4.3%)	22 (7.1%)	
Lymph node staging				0.47
Sentinel lymph node biopsy	50 (40.3%)	76 (41.3%)	126 (40.9%)	
Axillary lymph node dissection	73 (58.9%)	108 (58.7%)	181 (58.8%)	
None	1 (0.8%)	0 (0.0%)	1 (0.3%)	
Neoadjuvant chemotherapy				0.10
Yes	74 (59.7%)	91 (49.5%)	165 (53.6%)	
No	50 (40.3%)	93 (50.5%)	143 (46.4%)	
Adjuvant chemotherapy				0.11
Yes	59 (47.6%)	106 (57.6%)	165 (53.6%)	
No	65 (52.4%)	78 (42.4%)	143 (46.4%)	
Hormone therapy				0.38
Yes	93 (75.0%)	147 (79.9%)	240 (77.9%)	
No	31 (25.0%)	37 (20.1%)	68 (22.1%)	
Targeted therapy				0.72
Yes	32 (25.8%)	43 (23.4%)	75 (24.4%)	
No	92 (74.2%)	141 (76.6%)	233 (75.6%)	
Reconstruction stage				0.62
Immediate	20 (16.1%)	35 (19.0%)	55 (17.9%)	
two-stage delayed	104 (83.9%)	149 (81.0%)	253 (82.1%)	
Implant volume (cc) at the final reconstruction	408.3 ± 82.9	406.6 ± 86.8	407.3 ± 85.1	0.87
Implant volume (cc) at PMRT ^b^	360.4 ± 113.3	363.2 ± 99.3	362.1 ± 105.0	0.82
Interval between initial reconstruction and PMRT (weeks)	14.6 ± 11.6	18.3 ± 12.2	16.8 ± 12.1	0.01
RT technique				< 0.001
3D-CRT	31 (25.0%)	2 (1.1%)	33 (10.7%)	
IMRT	90 (72.6%)	136 (73.9%)	226 (73.4%)	
VMAT	3 (2.4%)	46 (25.0%)	49 (15.9%)	
EQD2 (Gy)	52.1 ± 4.5	50.6 ± 4.3	51.2 ± 4.4	0.003
RT to IMN				< 0.001
Yes	81 (65.3%)	168 (91.3%)	249 (80.8%)	
No	43 (34.7%)	16 (8.7%)	59 (19.2%)	
RT to SCV				0.71
Yes	104 (83.9%)	150 (81.5%)	254 (82.5%)	
No	20 (16.1%)	34 (18.5%)	54 (17.5%)	
Boost RT to tumor bed				1.00
Yes	10 (8.1%)	14 (7.6%)	24 (7.8%)	
No	114 (91.9%)	170 (92.4%)	284 (92.2%)	
Bolus				0.77
Yes	10 (8.1%)	12 (6.5%)	22 (7.1%)	
No	114 (91.9%)	172 (93.5%)	286 (92.9%)	

AJCC, American Joint Committee on Cancer; PMRT, postmastectomy radiotherapy; RT, radiation therapy; 3D-CRT, three-dimensional conformal RT; IMRT, intensity modulated radiotherapy; VMAT, volumetric modulated arc therapy; EQD2, equivalent dose in 2 Gy fractions; IMN, internal mammary nodes; SCV, supraclavicular volume.

a Clinical stage at the time of diagnosis.

b In the case of two-stage delayed reconstruction, inflated tissue expander volume at the initiation of PMRT was estimated.

### 3.2 Breast complications

The cumulative incidence of major breast complications at 1, 2, and 3 years were 6.6%, 10.3%, and 12.6%, respectively, in the ESTRO-T group and 9.7%, 15.4%, and 16.3%, respectively, in the CONV-T group. The incidence of any breast complications at 1, 2, and 3 years were 7.1%, 17.1%, and 18.9%, respectively, in the ESTRO-T group and 11.4%, 20.7%, and 23.3%, respectively, in the CONV-T group. From univariate analyses for major breast complications, body mass index (BMI) ≥ 23 [subdistribution hazard ratio (sHR), 1.91; P = 0.03)], clinical N2–3 (cN2–3) stage (sHR, 1.78; p = 0.04), skin invasion (sHR, 1.78; p = 0.11), targeted therapy (sHR, 0.52; P = 0.12), implant volume at the final reconstruction ≥ 450cc (sHR, 1.64; p = 0.10), the interval between reconstruction, and RT ≥ 7 weeks (sHR, 0.67; p = 0.17) were included in the multivariable analyses subsequently. However, none of these factors showed significance in the multivariable analyses including target volume delineation [sHR, 0.87; 95% confidence interval (CI), 0.46–1.68; p = 0.77) (**Table 2**). For any breast complications, smoking history (sHR, 3.47; 95% CI, 1.51–7.97; p = 0.03) and cN2–3 stage (sHR, 1.55; 95% CI, 1.01–2.38; p = 0.04) were significant adverse factors in the multivariable analyses. Target volume delineation was not significantly associated with any breast complication (sHR, 0.97; 95% CI, 0.55–1.70; p = 0.92). The cumulative incidences of major wound-related complications have no statistically significant differences between the two groups by 3 years (8.1% in the ESTRO-T group and 12.2% in the CONV-T group; p = 0.24). The 3-year major implant-related complications were also similar: 4.5% vs. 4.1%; p = 0.56. Cumulative incidences of any wound-related and implant-related complications showed no statistical differences (p = 0.06 and 0.18, respectively) (**Supplementary Figure 1**).

**Table 2:** Univariate and multivariable analyses of major breast complications and any breast complications in all patients.

	Major breast complications				Any breast complications			
	Univariate		Multivariable		Univariate		Multivariabe	
	HR (95% CI)	P–value	HR (95% CI)	P–value	HR (95% CI)	P–value	HR (95% CI)	P–value
Target volume delineation								
ESTRO-ACROP (vs. conventional)	0.82 (0.44–1.55)	0.56	0.87 (0.46–1.68)	0.69	0.86 (0.52–1.42)	0.56	0.97 (0.55–1.70)	0.92
Age (years)								
≥ 45 (vs. < 45)	0.78 (0.44–1.40)	0.41			0.92 (0.57–1.47)	0.72		
Diabetes mellitus								
Yes (vs. no)	0.63 (0.09–4.46)	0.65			0.95 (0.25–3.60)	0.07	0.45 (0.08–2.49)	0.36
Body mass index (kg/m^2^)								
≥ 23 (vs. < 23)	1.91 (1.06–3.43)	0.03	1.39 (0.72–2.66)	0.32	1.73 (1.07–2.79)	0.02	1.49 (0.88–2.51)	0.14
Smoking Hx								
Yes (vs. no)	1.73 (0.43–6.93)	0.44			3.82 (1.60–9.16)	0.003	3.47 (1.51–7.97)	0.003
T stage								
T3–4 (vs. T1–2)	1.26 (0.68–2.34)	0.46			1.22 (0.73–2.04)	0.45		
N stage								
N2–3 (vs. N0–1)	1.78 (1.04–3.03)	0.04	1.70 (0.98–2.95)	0.06	1.47 (0.98–2.20)	0.07	1.55 (1.01–2.38)	0.04
Skin invasion								
Yes (vs. no)	1.97 (0.85–4.55)	0.11	1.51 (0.64–3.57)	0.35	2.11 (1.03–4.33)	0.04	2.04 (0.91–4.57)	0.08
Neoadjuvant chemotherapy								
Yes (vs. no)	1.34 (0.74–2.43)	0.33			1.06 (0.66–1.70)	0.89		
Adjuvant chemotherapy								
Yes (vs. no)	0.75 (0.42–1.33)	0.32			0.97 (0.60–1.57)	0.91		
Hormone therapy								
Yes (vs. no)	1.01 (0.50–2.07)	0.97			1.34 (0.71–2.54)	0.37		
Targeted therapy								
Yes (vs. no)	0.52 (0.23–1.19)	0.12	0.44 (0.18–1.09)	0.08	0.78 (0.43–1.42)	0.41		
Reconstruction stage								
Immediate (vs. two-stage delayed)	1.24 (0.62–2.45)	0.54			1.02 (0.56–1.85)	0.95		
Implant volume (cc) at the final reconstruction								
≥ 450 (vs. < 450)	1.64 (0.91–2.96)	0.10	1.43 (0.76–2.69)	0.26	1.52 (0.94–2.46)	0.09	1.23 (0.73–2.08)	0.44
Implant volume (cc) at the PMRT								
≥ 350 (vs. < 350)	0.98 (0.55–1.75)	0.94			1.06 (0.65–1.71)	0.82		
Interval between initial reconstruction and PMRT (weeks)								
≥ 7 (vs. < 7)	0.67 (0.37–1.19)	0.17			0.82 (0.51–1.32)	0.42		
RT modality								
IMRT, VMAT (vs. 3D-CRT)	0.75 (0.43–1.31)	0.31			0.92 (0.56–1.53)	0.75		
EQD2 (Gy_3.5_)								
≥ 50 (vs. < 50)	0.92 (0.49–1.73)	0.80			0.74 (0.43–1.26)	0.26		
RT to IMN								
Yes (vs. no)	1.66 (0.65–4.22)	0.29			1.54 (0.74–3.23)	0.25		
RT to SCV								
Yes (vs. no)	0.73 (0.37–1.45)	0.37			0.64 (0.37–1.10)	0.11	0.58 (0.30–1.13)	0.11
Boost RT to tumor bed								
Yes (vs. no)	0.54 (0.13–2.22)	0.39			0.54 (0.17–1.73)	0.30		

HR, hazard ratio; 95% CI, 95% confidence interval.

We conducted additional subgroup analyses on the two types of reconstruction: two-stage delayed reconstruction and immediate reconstruction. Patient characteristics of each two types are shown in **Supplementary Tables 1, 2**. Among 253 patients with two-stage delayed reconstruction with a temporary tissue expander, the 1-, 2-, and 3-year rates of all major breast complications were 6.7%, 9.9%, and 12.9% in the ESTRO-T group, respectively, and 10.6%, 14.5%, and 15.5% in the CONV-T group, respectively (p = 0.64). The incidence of any breast complications at 3 years was 19.0% among ESTRO-T group and 22.9% among CONV-T group (p = 0.63) (**Supplementary Figures 2A, B**). The differences in both major wound-related complications (sHR, 0.67; 95% CI, 0.31–1.46; p = 0.32) and implant-related complications (sHR, 1.92; 95% CI, 0.37–9.91; p = 0.43) were not significant by three years after PMRT.

For 55 patients who received immediate reconstruction with a permanent implant, there was no significant difference in the cumulative incidences of major breast complications between the ESTRO-T group and CONV-T group at 1, 2, and 3 years (5.7%, 11.6%, and 11.6% vs. 5.0%, 20.1%, and 20.1%; p = 0.71). All any breast complications were not significantly different with the 3-year cumulative incidence of 18.2% and 25.6%, respectively (p = 0.77) (**Supplementary Figures 2C, D**). No significant difference was shown in the occurrence of wound-related complications (sHR, 0.56; 95% CI, 0.04–8.65; p = 0.68) and implant-related complications (sHR, 0.85; 95% CI, 0.18–4.09; p = 0.84) between two groups.

### 3.3 Radiation-induced toxicities

Symptomatic radiation-induced pneumonitis was developed in six (3.2%) and three (2.4%) patients in the ESTRO-T and CONV-T groups, with a mean interval of 6.0 months from completion of PMRT. Among these patients, three (1.6%) and three (2.4%) patients in each group experienced grade 2 pneumonitis and were treated with oral medication. Other patients had minor clinical symptoms and did not require medical intervention. There was no significant difference in the incidence of radiation-induced pneumonitis (p = 0.68). No more than grade 3 pneumonitis was observed during the follow-up. Furthermore, no adverse events to the heart were reported. **Supplementary Table 3** summarized quantitative dosimetric analyses for the ipsilateral lung and heart.

### 3.4 Locoregional control

In our cohort, only one patient in the ESTRO-T group experienced a locoregional recurrence 18.9 months after the completion of PMRT. This recurrence was located in the chest wall ventral to the implant, and within the target field based on both the conventional and the ESTRO-ACROP target volume delineation guidelines. No locoregional recurrence was found in patients in the CONV-T group.

## 4 Discussion

For patients with breast cancer, breast reconstruction after mastectomy has gained greater prominence as a means of preserving the patient body image and overall psychosocial recovery (20). Indeed, contemporary studies suggest a substantial escalation in the number of patients receiving PMRT and breast reconstruction, and implant-based methods have predominantly been used (21). A recent population-based research reported nearly three-quarters of the patients received implant-based reconstruction (22). However, breast reconstruction can be challenging when integrating with PMRT, which may compromise the skin and underlying tissue due to radiation toxicity, including skin changes, vascular compromise, and fibrosis (23, 24), which can compromise the viability and cosmesis of the reconstruction and may require repeated intervention (10).

The new ESTRO-ACROP guideline is expected to serve as a template for establishing target volume for PMRT after implant-based reconstruction, and it is necessary to evaluate oncological outcomes, treatment safety, and cosmetic outcomes of patients who are treated according to this guideline. The present study analyzed 308 patients diagnosed with breast cancer who underwent implant-based subpectoral breast reconstruction and PMRT with the new 2019 ESTRO-ACROP target volume delineation or conventional target volume delineation. We found that the new updated ESTRO-ACROP target volume delineation did not affect breast complications compared to conventional target volume delineation, including wound-related and implant-related complications. Multivariable analyses revealed no statistically significant associations with the incidence of major breast complications. However, smoking history and advanced N stage were significant risk factors for any breast complications. It is noteworthy that, while it did not reach statistical significance, there was a consistent trend indicating that breast complications decreased with the implementation of ESTRO-ACROP target volume delineation guidelines.

For patients who are going to receive implant-based reconstruction, the timing of reconstruction may have an impact on breast complications. Several studies including meta-analyses, comparing the outcomes of two-stage and immediate reconstruction, showed that PMRT on the tissue expander can reduce the risk of severe capsular contracture but aggravate reconstruction failure compared to PMRT on the permanent implant (24–26). However, another recent study showed that RT after direct-to-implant breast reconstruction had a lower rate of complications and reconstructive failure compared to tissue expander-implant reconstruction (27). Moreover, Naoum et al. revealed similar results that complication rates of the single-stage implant were not significantly different from autologous reconstruction and lower than two-stage tissue expander-based reconstruction in PMRT settings (28). In the current study, reconstruction timing did not show a significant association with breast complications, and the ESTRO-ACROP target volume delineation method did not affect complications in either two-stage delayed reconstruction or immediate reconstruction subgroups. For implant placement, the differences in breast complications between prepectoral and subpectoral approaches are controversial yet (29–31). We do find it reassuring that the rates of breast complications observed in our cohort were generally comparable to those reported in previous studies. Our findings suggest that introducing the new ESTRO-ACROP guideline is feasible for patients who underwent subpectoral reconstruction in terms of breast complications.

Based on well-known randomized trials that established hypofractionated regimen as an effective alternative for adjuvant RT after breast-conserving surgery and mastectomy (32–35), a multi-institutional study by the Korean Radiation Oncology Group evaluated the feasibility of hypofractionated RT after breast reconstruction. It revealed that hypofractionated PMRT can improve breast reconstruction outcomes (36). Other recent retrospective studies also suggested that a hypofractionated regimen was comparable with a conventional fractionation in terms of breast-related complications, regardless of breast reconstruction type (14) and surgical extent (37).

The major difference between the conventional and the 2019 ESTRO-ACROP guidelines is in the definition of the CTV of the chest wall. Whereas prior contouring guidelines generally included the whole implant, the new ESTRO-ACROP guidelines removed it from the CTV in selected patients (16, 18). Of note, in patients with subpectoral implant breast reconstruction, where implants were inserted in the pocket between the pectoral major and minor, a convex strip of subcutaneous and remnant breast tissue between the anterior and skin of the pectoral major was covered.

The new ESTRO-ACROP guideline has dosimetric benefits to adjacent normal organs when using modern volume-based planning techniques. Chang et al. compared dosimetric characteristics of patients with left-sided breast cancer between two guidelines in VMAT planning. It revealed that the new target volume delineation method significantly reduced exposure to the heart, left anterior descending coronary artery (LAD), and ipsilateral lung, maintaining target coverage, delivery accuracy, and dose heterogeneity compared with conventional delineation (17). Similarly, Milligan et al. also evaluated the changes in normal organ sparing and target coverage with VMAT and pencil-beam scan planning, finding that the ESTRO target has dosimetric advantages to cardiopulmonary organs (18). Previous studies have shown that increasing radiation doses to the heart, left ventricle, and LAD are directly associated with long-term rates of high-grade coronary artery stenosis and acute coronary events (38–40). Also, radiation pneumonitis and radiation fibrosis are well-known toxicities caused by RT in patients with breast cancer, which have a correlation with increasing radiation dose to the lung (41, 42). It is noteworthy that the new guideline could minimize RT-induced adverse events, as most patients with breast cancer are expected to have long-term survival.

There might be a concern about increasing recurrences at deep chest wall structures, which were excluded from the target volume in the new guideline. It is based on location-specific recurrence patterns as well as residual glandular tissues and draining lymphatics from surgical data (43, 44). The first study that identified the locoregional recurrence patterns according to the anatomical sites analyzed 1,571 mastectomy patients and conducted a systematic review of 14 publications. Only one relapse developed in the deep chest wall structures (pectoral minor, intercostal muscle, and rib), which is not regarded as the irradiated volume in the present guideline. Meta-analysis also reported the paucity of recurrences to deep chest wall compared with those to skin/subcutaneous and pectoral muscle (45). Joo et al. also analyzed the three-dimensional location and pattern of local recurrence of 51 patients who underwent implant-based breast reconstruction and presented ipsilateral breast tumor recurrences. Of all recurrent tumors, 7% developed in the pectoral major and deep thoracic muscle in subpectoral implant patients. No recurrence occurred in the implant pocket (46). These analyses suggest that the application of the ESTRO-ACROP consensus guideline is feasible in regard to chest wall recurrence. However, they analyzed patients who had received adjuvant RT before the ESTRO-ACROP guideline was established, so an accurate comparison to the effect of the updated guideline was not performed. Further studies should be needed to aid in data collection, analyses, and clinical decision-making.

We have several limitations to this study. As this is a retrospective study that is based on a medical chart data review, there is a possibility of selection bias. We attempted to mitigate this by adjusting for multiple risk factors in the multivariable analyses. In this study design, the patients who had adverse factors (T4 disease, poor response to chemotherapy, invasion of the pectoral muscle or deeper structures, and inflammatory breast cancer) followed conventional guidelines to cover enough dorsal portion of the implant and the chest wall. It may explain that patients in the CONV-T group had higher T stage (T3–4) than those in the ESTRO-T group (41.1 % vs. 25.5 %; p= .006). However, T stage was not significantly associated with major and any breast complications in the univariate and multivariable analyses. Follow-up duration was significantly shorter in the ESTRO-T group (median, 29.4 months) than in the CONV-T group (median, 46.8 months), as we implemented the new ESTRO-ACROP delineation guideline for treatment in June 2018, following an open panel discussion held during the ESTRO 2018 conference (47, 48). The higher prevalence of IMN irradiation and VMAT planning in the ESTRO-T group can be attributed to the changes in treatment strategies in our institutions. For example, IMN irradiation has been introduced for selective node-positive patients undergoing regional nodal irradiation, with evidence of survival improvement (49, 50). Also, we adopted VMAT technologies as the standard for PMRT planning in 2020 to optimize dose distribution with better homogeneity and reduce radiation-related pneumonitis (51). Lastly, our follow-up duration can be relatively short to comprehensively evaluate breast-related complications after reconstruction and PMRT over time (9, 10). Therefore, for this study, the endpoint was defined as complications occurring within 3 years rather than encompassing “all late complications.” Patients with a follow-up duration of less than 6 months were excluded to ensure a more accurate evaluation of events within the specified 3-year timeframe. Given the publication of the new ESTRO-ACROP guideline in 2019, the follow-up time was significantly shorter in the ESTRO-T group, which could potentially lead to an underestimation of event rates. Further monitoring will be necessary to validate the long-term efficacy and safety such as radiation-induced coronary artery diseases, which are known as a long-term effect of radiotherapy for left-sided breast cancer.

To date, this is the first study to report breast complication outcomes in patients with breast cancer who received PMRT with the new ESTRO-ACROP guideline. Our results indicate that target volume delineation according to the new ESTRO-ACROP guideline showed no significant differences compared to the conventional target volume delineation in regard to breast complications in the first 3 years from PMRT. It also demonstrated comparable radiation-induced pneumonitis and locoregional control. As the use of the new recommendations showed dosimetric benefits of OARs and did not result in increased risk of locoregional recurrences, long-term follow-up analyses and randomized clinical trials, such as the ongoing DBCG-RT phase III trial, are necessary to evaluate further clinical outcomes.

## Data availability statement

The raw data supporting the conclusions of this article will be made available by the authors, without undue reservation.

## Ethics statement

The studies involving humans were approved by IRB number: H-2204-102-1316, B-2206-760-401. The studies were conducted in accordance with the local legislation and institutional requirements. The participants provided their written informed consent to participate in this study.

## Author contributions

JP: Data curation, Formal analysis, Investigation, Methodology, Resources, Software, Validation, Visualization, Writing – original draft, Writing – review & editing. B-SJ: Investigation, Writing – review & editing, Resources. JC: Conceptualization, Investigation, Methodology, Project administration, Resources, Writing – review & editing. JK: Investigation, Resources, Writing – review & editing. CC: Investigation, Software, Writing – review & editing. KH: Methodology, Resources, Writing – review & editing. UJ: Methodology, Resources, Writing – review & editing. HC: Methodology, Resources, Writing – review & editing. YM: Methodology, Resources, Writing – review & editing. JJ: Writing – review & editing, Methodology, Resources. CH: Methodology, Resources, Writing – review & editing. IK: Methodology, Resources, Writing – review & editing. KS: Conceptualization, Funding acquisition, Investigation, Methodology, Project administration, Supervision, Validation, Writing – review & editing, Resources.
