# Supplementary material for: The impact of the new ESTRO-ACROP target volume delineation guidelines for postmastectomy radiotherapy after implant-based breast reconstruction on breast complications
Source: Front Oncol. 2024 May 23;14:1373434. doi: 10.3389/fonc.2024.1373434 (PMC11153655; doi:10.3389/fonc.2024.1373434)
Supplement: Supplementary file 1 [file Image_1.pdf]

Supplementary Material

Supplementary Figures

(A) (B)

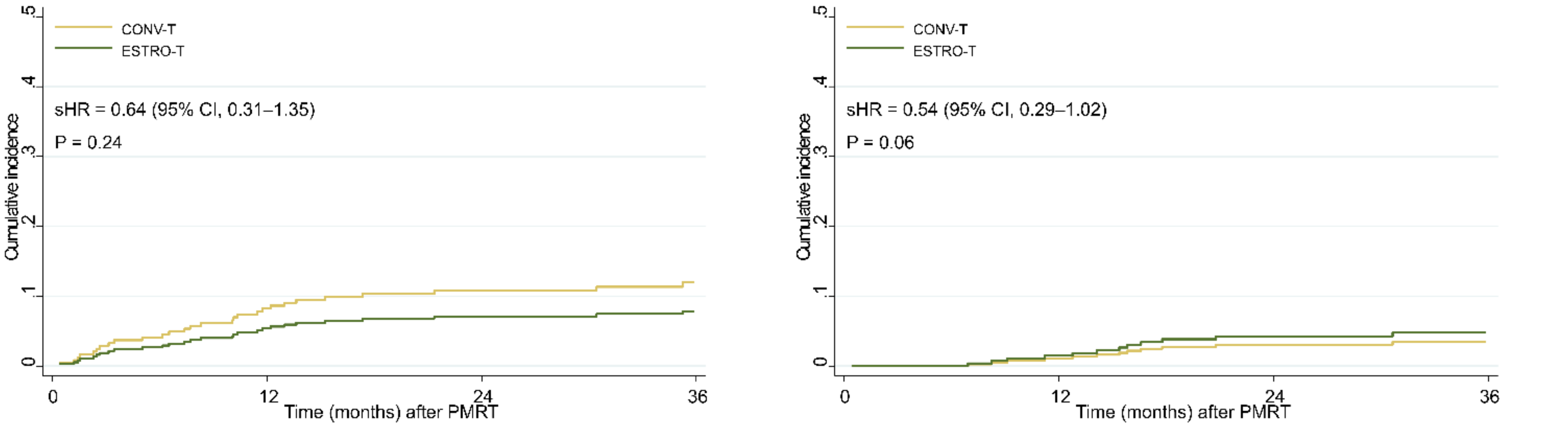

Supplementary Figure 1. Cumulative incidence curves of (A) major wound-related complications and (B) major implant-related complications in all patients.
