# Supplementary material for: The impact of the new ESTRO-ACROP target volume delineation guidelines for postmastectomy radiotherapy after implant-based breast reconstruction on breast complications
Source: Front Oncol. 2024 May 23;14:1373434. doi: 10.3389/fonc.2024.1373434 (PMC11153655; doi:10.3389/fonc.2024.1373434)
Supplement: Supplementary file 2 [file Image_2.pdf]

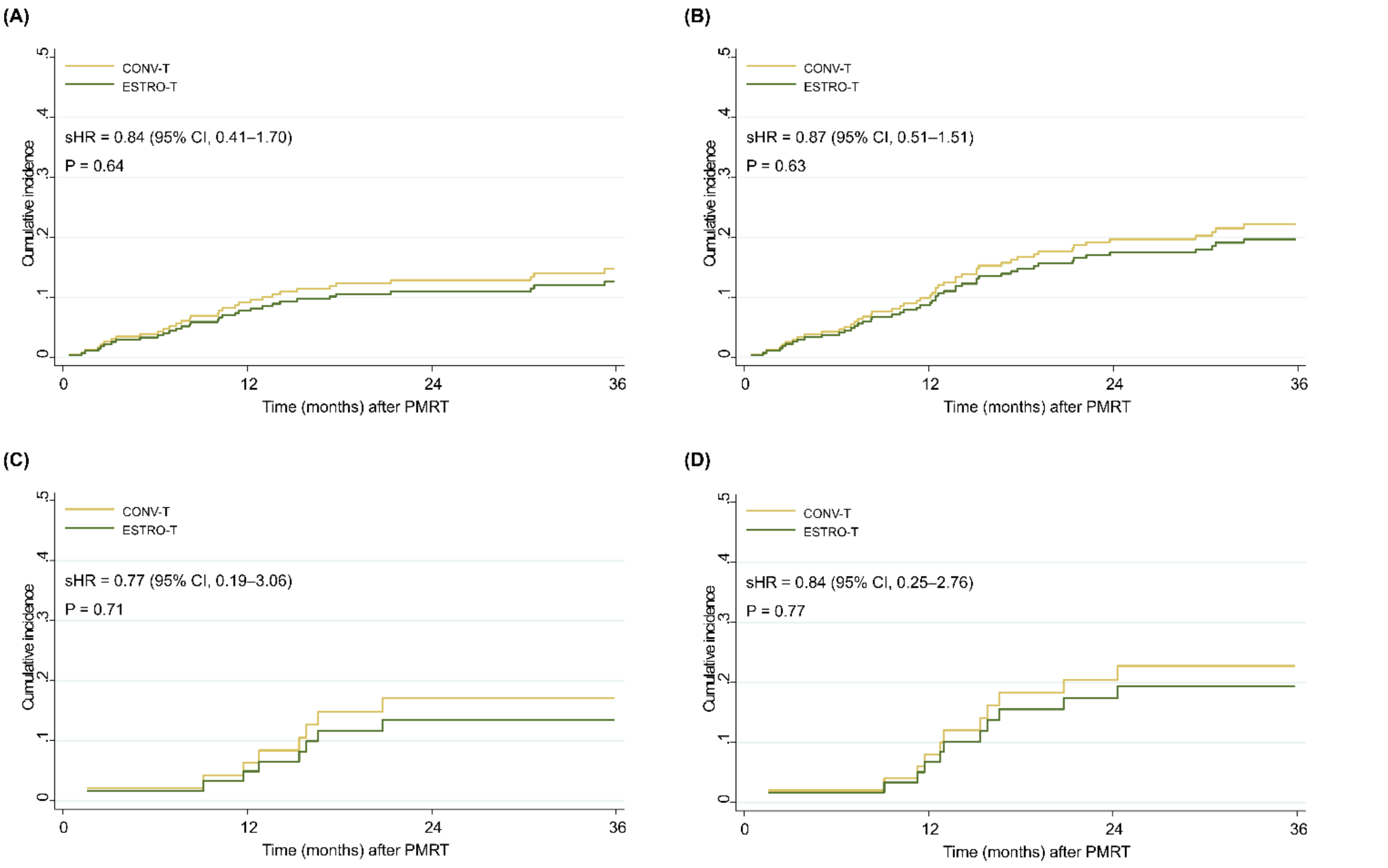

Supplementary Figure 2. Cumulative incidence curves of (A) major breast complications and (B) any breast complications in patients undergone two-stage delayed reconstruction, and (C) major breast complications and (D) any breast complications in patients undergone immediate reconstruction.
