# Supplementary material for: The impact of the new ESTRO-ACROP target volume delineation guidelines for postmastectomy radiotherapy after implant-based breast reconstruction on breast complications
Source: Front Oncol. 2024 May 23;14:1373434. doi: 10.3389/fonc.2024.1373434 (PMC11153655; doi:10.3389/fonc.2024.1373434)
Supplement: Supplementary file 3 [file Table_1.docx]

Supplementary Material

# Supplementary Tables

**Supplementary Table 1.** Patient characteristics in patients undergoing two-stage delayed reconstruction

|  | **CONV-T (N=104)** | **ESTRO-T**  **(N=149)** | **Total**  **(N=253)** | **P value** |
| --- | --- | --- | --- | --- |
| Age (years), mean | 45.3 ± 7.8 | 46.6 ± 8.2 | 46.1 ± 8.1 | 0.18 |
| Age (years) |  |  |  | 0.10 |
| ≥ 45 | 53 (51.0%) | 59 (39.6%) | 112 (44.3%) |  |
| < 45 | 51 (49.0%) | 90 (60.4%) | 141 (55.7%) |  |
| Follow-up duration (months) | 46.5 ± 21.8 | 28.5 ± 13.4 | 35.9 ± 19.5 | < 0.001 |
| Diabetes mellitus |  |  |  | 0.89 |
| Yes | 3 (2.9%) | 6 (4.0%) | 9 (3.6%) |  |
| No | 101 (97.1%) | 143 (96.0%) | 244 (96.4%) |  |
| Body mass index (kg/m^2^) |  |  |  | 0.90 |
| < 23 | 61 (58.7%) | 85 (57.0%) | 146 (57.7%) |  |
| ≥ 23 | 43 (41.3%) | 64 (43.0%) | 107 (42.3%) |  |
| Smoking history |  |  |  | 1.00 |
| Yes | 2 (1.9%) | 4 (2.7%) | 6 (2.4%) |  |
| No | 102 (98.1%) | 145 (97.3%) | 247 (97.6%) |  |
| Laterality |  |  |  | 0.34 |
| Left | 63 (60.6%) | 80 (53.7%) | 143 (56.5%) |  |
| Right | 41 (39.4%) | 69 (46.3%) | 110 (43.5%) |  |
| Histologic type |  |  |  | 0.58 |
| Intraductal carcinoma | 86 (82.7%) | 128 (85.9%) | 214 (84.6%) |  |
| Intralobular carcinoma | 12 (11.5%) | 12 (8.1%) | 24 (9.5%) |  |
| Others | 6 (5.8%) | 9 (6.0%) | 15 (5.9%) |  |
| T stage (AJCC 8th) ^a^ |  |  |  | 0.18 |
| T1 | 9 (8.7%) | 19 (12.8%) | 28 (11.1%) |  |
| T2 | 51 (49.0%) | 86 (57.7%) | 137 (54.2%) |  |
| T3 | 31 (29.8%) | 33 (22.1%) | 64 (25.3%) |  |
| T4 | 13 (12.5%) | 11 (7.4%) | 24 (9.5%) |  |
| N stage (AJCC 8^th^) ^a^ |  |  |  | 0.44 |
| N0 | 12 (11.5%) | 10 (6.7%) | 22 (8.7%) |  |
| N1 | 53 (51.0%) | 72 (48.3%) | 125 (49.4%) |  |
| N2 | 27 (26.0%) | 44 (29.5%) | 71 (28.1%) |  |
| N3 | 12 (11.5%) | 23 (15.4%) | 35 (13.8%) |  |
| Molecular type |  |  |  | 0.46 |
| Luminal A | 62 (59.6%) | 82 (55.0%) | 144 (56.9%) |  |
| Luminal B1 | 5 (4.8%) | 16 (10.7%) | 21 (8.3%) |  |
| Luminal B2 | 13 (12.5%) | 18 (12.1%) | 31 (12.3%) |  |
| HER-2 enriched | 11 (10.6%) | 19 (12.8%) | 30 (11.9%) |  |
| Triple negative breast cancer | 13 (12.5%) | 14 (9.4%) | 27 (10.7%) |  |
| Skin invasion |  |  |  | 0.69 |
| Yes | 13 (12.5%) | 15 (10.1%) | 28 (11.1%) |  |
| No | 91 (87.5%) | 134 (89.9%) | 225 (88.9%) |  |
| Nipple invasion |  |  |  | 1.00 |
| Yes | 14 (13.5%) | 20 (13.4%) | 34 (13.4%) |  |
| No | 90 (86.5%) | 129 (86.6%) | 219 (86.6%) |  |
| Muscle invasion |  |  |  | 0.19 |
| Yes | 4 (3.8%) | 1 (0.7%) | 5 (2.0%) |  |
| No | 100 (96.2%) | 148 (99.3%) | 248 (98.0%) |  |
| Mastectomy |  |  |  | 0.21 |
| Nipple-sparing mastectomy | 11 (10.6%) | 12 (8.1%) | 23 (9.1%) |  |
| Skin-sparing mastectomy | 82 (78.8%) | 129 (86.6%) | 211 (83.4%) |  |
| Total mastectomy | 11 (10.6%) | 8 (5.4%) | 19 (7.5%) |  |
| Lymph node staging |  |  |  | 0.35 |
| Sentinel lymph node biopsy | 37 (35.6%) | 61 (40.9%) | 98 (38.7%) |  |
| Axillary lymph node dissection | 66 (63.5%) | 88 (59.1%) | 154 (60.9%) |  |
| None | 1 (1.0%) | 0 (0.0%) | 1 (0.4%) |  |
| Neoadjuvant chemotherapy |  |  |  | 0.28 |
| Yes | 59 (56.7%) | 73 (49.0%) | 132 (52.2%) |  |
| No | 45 (43.3%) | 76 (51.0%) | 121 (47.8%) |  |
| Adjuvant chemotherapy |  |  |  | 0.25 |
| Yes | 53 (51.0%) | 88 (59.1%) | 141 (55.7%) |  |
| No | 51 (49.0%) | 61 (40.9%) | 112 (44.3%) |  |
| Hormone therapy |  |  |  | 0.69 |
| Yes | 80 (76.9%) | 119 (79.9%) | 199 (78.7%) |  |
| No | 24 (23.1%) | 30 (20.1%) | 54 (21.3%) |  |
| Targeted therapy |  |  |  | 1.00 |
| Yes | 26 (25.0%) | 36 (24.2%) | 62 (24.5%) |  |
| No | 78 (75.0%) | 113 (75.8%) | 191 (75.5%) |  |
| Implant volume (cc) at the final reconstruction | 416.4 ± 82.1 | 417.8. ± 83.1 | 417.2 ± 82.5 | 0.90 |
| Implant volume (cc) at PMRT ^b^ | 360.1 ± 119.9 | 364.7 ± 102.3 | 362.8 ± 109.7 | 0.74 |
| Interval between initial reconstruction and PMRT (weeks) | 15.9 ± 11.9 | 18.6 ± 12.2 | 17.5 ± 12.1 | 0.08 |
| RT technique |  |  |  | < 0.001 |
| 3D-CRT | 28 (26.9%) | 2 (1.3%) | 30 (11.9%) |  |
| IMRT | 74 (71.2%) | 107 (71.8%) | 181 (71.5%) |  |
| VMAT | 2 (1.9%) | 40 (26.8%) | 42 (16.6%) |  |
| EQD2 (Gy) | 52.1 ± 4.7 | 50.8 ± 4.4 | 51.3 ± 4.6 | 0.03 |
| RT to IMN |  |  |  | < 0.001 |
| Yes | 67 (64.4%) | 141 (94.6%) | 208 (82.2%) |  |
| No | 37 (35.6%) | 8 (5.4%) | 45 (17.8%) |  |
| RT to SCV |  |  |  | 0.79 |
| Yes | 88 (84.6%) | 123 (82.6%) | 211 (83.4%) |  |
| No | 16 (15.4%) | 26 (17.4%) | 42 (16.6%) |  |
| Boost RT |  |  |  | 1.00 |
| Yes | 8 (7.7%) | 12 (8.1%) | 20 (7.9%) |  |
| No | 96 (92.3%) | 137 (91.9%) | 233 (92.1%) |  |
| Bolus |  |  |  | 0.74 |
| Yes | 9 (8.7%) | 10 (6.7%) | 19 (7.5%) |  |
| No | 95 (91.3%) | 139 (93.3%) | 234 (92.5%) |  |

Abbreviations: AJCC = American Joint Committee on Cancer; PMRT = postmastectomy radiotherapy; RT = radiotherapy; 3D-CRT = 3-dimensional conformal RT; IMRT = intensity modulated radiotherapy; VMAT = volumetric modulated arc therapy; EQD2 = equivalent dose in 2 Gy fractions; IMN = internal mammary nodes; SCV = supraclavicular volume

^a^ clinical stage at the time of diagnosis

^b^ In the case of two-stage delayed reconstruction, inflated tissue expander volume at the initiation of PMRT was estimated
