# Supplementary material for: The impact of the new ESTRO-ACROP target volume delineation guidelines for postmastectomy radiotherapy after implant-based breast reconstruction on breast complications
Source: Front Oncol. 2024 May 23;14:1373434. doi: 10.3389/fonc.2024.1373434 (PMC11153655; doi:10.3389/fonc.2024.1373434)
Supplement: Supplementary file 4 [file Table_2.docx]

Supplementary Material

# Supplementary Tables

**Supplementary Table 2.** Patient characteristics in patients undergoing immediate reconstruction

|  | **CONV-T (N=20)** | | **ESTRO-T**  **(N=35)** | | **Total**  **(N=55)** | **P value** |
| --- | --- | --- | --- | --- | --- | --- |
| Age (years), mean | 43.5 ± 8.4 | | 45.3 ± 8.0 | | 44.6 ± 8.1 | 0.42 |
| Age (years) |  | |  | |  | 1.00 |
| ≥ 45 | 10 (50.0%) | | 17 (48.6%) | | 27 (49.1%) |  |
| < 45 | 10 (50.0%) | | 18 (51.4%) | | 28 (50.9%) |  |
| Follow-up duration (months) | 48.6 ± 26.6 | | 33.2 ± 13.0 | | 38.8 ± 20.3 | 0.02 |
| Diabetes mellitus |  | |  | |  | 1.00 |
| Yes | 20 (100.0%) | | 34 (97.1%) | | 54 (98.2%) |  |
| No | 0 (0.0%) | | 1 (2.9%) | | 1 (1.8%) |  |
| Body mass index (kg/m^2^) |  | |  | |  | 0.98 |
| < 23 | 10 (50.0%) | | 16 (45.7%) | | 26 (47.3%) |  |
| ≥ 23 | 10 (50.0%) | | 19 (54.3%) | | 29 (52.7%) |  |
| Smoking history |  | |  | |  | 1.00 |
| Yes | 1 (5.0%) | | 2 (5.7%) | | 3 (5.5%) |  |
| No | 19 (95.0%) | | 33 (94.3%) | | 52 (94.5%) |  |
| Laterality |  | |  | |  | 0.15 |
| Left | 14 (70.0%) | | 16 (45.7%) | | 30 (54.5%) |  |
| Right | 6 (30.0%) | | 19 (54.3%) | | 25 (45.5%) |  |
| Histologic type |  | |  | |  | 0.45 |
| Intraductal carcinoma | 17 (85.0%) | | 32 (91.4%) | | 49 (89.1%) |  |
| Intralobular carcinoma | 2 (10.0%) | | 2 (5.7%) | | 4 (7.3%) |  |
| Others | 1 (5.0%) | | 1 (2.9%) | | 2 (3.6%) |  |
| T stage (AJCC 8th) ^a^ |  | |  | |  | 0.08 |
| T1 | 1 (5.0%) | | 5 (14.3%) | | 6 (10.9%) |  |
| T2 | 12 (60.0%) | | 27 (77.1%) | | 39 (70.9%) |  |
| T3 | 6 (30.0%) | | 2 (5.7%) | | 8 (14.5%) |  |
| T4 | 1 (5.0%) | | 1 (2.9%) | | 2 (3.6%) |  |
| N stage (AJCC 8^th^) ^a^ |  | |  | |  | 0.01 |
| N0 | 3 (15.0%) | | 2 (5.7%) | | 5 (9.1%) |  |
| N1 | 4 (20.0%) | | 23 (65.7%) | | 27 (49.1%) |  |
| N2 | 10 (50.0%) | | 7 (20.0%) | | 17 (30.9%) |  |
| N3 | 3 (15.0%) | | 3 (8.6%) | | 6 (10.9%) |  |
| Molecular type |  | |  | |  | 0.36 |
| Luminal A | 8 (40.0%) | | 19 (54.3%) | | 27 (49.1%) |  |
| Luminal B1 | 1 (5.0%) | | 3 (8.6%) | | 4 (7.3%) |  |
| Luminal B2 | 2 (10.0%) | | 6 (17.1%) | | 8 (14.5%) |  |
| HER-2 enriched | 4 (20.0%) | | 2 (5.7%) | | 6 (10.9%) |  |
| Triple negative breast cancer | 5 (25.0%) | | 5 (14.3%) | | 10 (18.2%) |  |
| Skin invasion |  | |  | |  | 0.78 |
| Yes | 1 (5.0%) | | 0 (0.0%) | | 1 (1.8%) |  |
| No | 19 (95.0%) | | 35 (100.0%) | | 54 (98.2%) |  |
| Nipple invasion |  | |  | |  | 0.78 |
| Yes | 1 (5.0%) | | 0 (0.0%) | | 1 (1.8%) |  |
| No | 19 (95.0%) | | 35 (100.0%) | | 54 (98.2%) |  |
| Muscle invasion |  | |  | |  | 0.78 |
| Yes | 1 (5.0%) | | 0 (0.0%) | | 1 (1.8%) |  |
| No | 19 (95.0%) | | 35 (100.0%) | | 54 (98.2%) |  |
| Mastectomy |  | |  | |  | 0.06 |
| Nipple-sparing mastectomy | 13 (65.0%) | | 27 (77.1%) | | 40 (72.7%) |  |
| Skin-sparing mastectomy | 4 (20.0%) | | 8 (22.9%) | | 12 (21.8%) |  |
| Total mastectomy | 3 (15.0%) | | 0 (0.0%) | | 3 (5.5%) |  |
| Lymph node staging |  | |  | |  | 0.19 |
| Sentinel lymph node biopsy | 13 (65.0%) | | 15 (42.9%) | | 28 (50.9%) |  |
| Axillary lymph node dissection | 7 (35.0%) | | 20 (57.1%) | | 27 (49.1%) |  |
| Neoadjuvant chemotherapy |  |  | |  | | 0.15 |
| Yes | 15 (75.0%) | 18 (51.4%) | | 33 (60.0%) | |  |
| No | 5 (25.0%) | 17 (48.6%) | | 22 (40.0%) | |  |
| Adjuvant chemotherapy |  |  | |  | | 0.21 |
| Yes | 6 (30.0%) | 18 (51.4%) | | 24 (43.6%) | |  |
| No | 14 (70.0%) | 17 (48.6%) | | 31 (56.4%) | |  |
| Hormone therapy |  |  | |  | | 0.37 |
| Yes | 13 (65.0%) | 28 (80.0%) | | 41 (74.5%) | |  |
| No | 7 (35.0%) | 7 (20.0%) | | 14 (25.5%) | |  |
| Targeted therapy |  |  | |  | | 0.61 |
| Yes | 6 (30.0%) | 7 (20.0%) | | 13 (23.6%) | |  |
| No | 14 (70.0%) | 28 (80.0%) | | 42 (76.4%) | |  |
| Implant volume (cc) at the final reconstruction | 366.0 ± 75.6 | 359.1 ± 87.1 | | 361.6 ± 82.5 | | 0.77 |
| Interval between initial reconstruction and PMRT (weeks) | 8.3 ± 7.7 | 17.1 ± 12.5 | | 13.9 ± 11.7 | | 0.002 |
| RT technique |  |  | |  | | 0.03 |
| 3D-CRT | 3 (15.0%) | 0 (0.0%) | | 3 (5.5%) | |  |
| IMRT | 16 (80.0%) | 29 (82.9%) | | 45 (81.8%) | |  |
| VMAT | 1 (5.0%) | 6 (17.1%) | | 7 (12.7%) | |  |
| EQD2 (Gy) | 52.3 ± 3.3 | 49.8 ± 3.3 | | 50.7 ± 3.5 | | 0.01 |
| RT to IMN |  |  | |  | | 0.79 |
| Yes | 14 (70.0%) | 27 (77.1%) | | 41 (74.5%) | |  |
| No | 6 (30.0%) | 8 (22.9%) | | 14 (25.5%) | |  |
| RT to SCV |  |  | |  | | 1.00 |
| Yes | 16 (80.0%) | 27 (77.1%) | | 43 (78.2%) | |  |
| No | 4 (20.0%) | 8 (22.9%) | | 12 (21.8%) | |  |
| Boost RT |  |  | |  | | 0.96 |
| Yes | 2 (10.0%) | 2 (5.7%) | | 4 (7.3%) | |  |
| No | 18 (90.0%) | 33 (94.3%) | | 51 (92.7%) | |  |
| Bolus |  |  | |  | | 1.00 |
| Yes | 1 (5.0%) | 2 (5.7%) | | 3 (5.5%) | |  |
| No | 19 (95.0%) | 33 (94.3%) | | 52 (94.5%) | |  |
