# Supplementary material for: The impact of the new ESTRO-ACROP target volume delineation guidelines for postmastectomy radiotherapy after implant-based breast reconstruction on breast complications
Source: Front Oncol. 2024 May 23;14:1373434. doi: 10.3389/fonc.2024.1373434 (PMC11153655; doi:10.3389/fonc.2024.1373434)
Supplement: Supplementary file 5 [file Table_3.docx]

Supplementary Material

# Supplementary Tables

**Supplementary Table 3.** Dosimetric analyses of the ipsilateral lung and the heart

| **Laterality** | **All** | | | **Left** | | | **Right** | | |
| --- | --- | --- | --- | --- | --- | --- | --- | --- | --- |
|  | **CONV-T**  **(N=119)** | **ESTRO-T**  **(N=173)** | P–value | **CONV-T**  **(N=74)** | **ESTRO-T**  **(N=92)** | P–value | **CONV-T**  **(N=45)** | **ESTRO-T**  **(N=81)** | P–value |
| Lung (Ipsilateral) |  |  |  |  |  |  |  |  |  |
| V5Gy ^a^ (%) | 73.9 ± 26.8 | 89.1 ± 10.1 | < 0.001 | 76.8 ± 23.7 | 84.9 ± 11.7 | 0.005 | 69.3 ± 31.1 | 94.0 ± 4.6 | < 0.001 |
| V10Gy (%) | 52.2 ± 21.2 | 55.5 ± 12.1 | 0.10 | 54.3 ± 18.2 | 52.2 ± 12.4 | 0.40 | 49.0 ± 25.3 | 59.2 ± 10.6 | 0.002 |
| V15Gy (%) | 36.8 ± 13.8 | 34.0 ± 7.3 | 0.03 | 38.1 ± 11.8 | 32.2 ± 7.9 | < 0.001 | 34.5 ± 16.4 | 36.1 ± 6.0 | 0.44 |
| V20Gy (%) | 27.7 ± 9.5 | 21.8 ± 5.7 | < 0.001 | 28.5 ± 8.1 | 20.7 ± 5.9 | < 0.001 | 26.5 ± 11.3 | 22.9 ± 5.2 | 0.02 |
| V25Gy (%) | 21.7 ± 7.4 | 12.8 ± 4.9 | < 0.001 | 22.1 ± 6.6 | 12.8 ± 5.0 | < 0.001 | 21.0 ± 8.7 | 12.9 ± 4.8 | < 0.001 |
| V30Gy (%) | 16.6 ± 6.3 | 6.8 ± 3.8 | < 0.001 | 16.9 ± 5.9 | 7.0 ± 4.0 | < 0.001 | 16.2 ± 6.9 | 6.6 ± 3.5 | < 0.001 |
| V35Gy (%) | 11.4 ± 5.5 | 3.1 ± 2.6 | < 0.001 | 11.5 ± 5.3 | 3.2 ± 2.9 | < 0.001 | 11.3 ± 5.9 | 2.9 ± 2.1 | < 0.001 |
| V40Gy (%) | 5.5 ± 4.3 | 0.9 ± 1.2 | < 0.001 | 5.4 ± 4.1 | 1.1 ± 1.5 | < 0.001 | 5.8 ± 4.6 | 0.9 ± 0.8 | < 0.001 |
| Mean dose (Gy) | 14.7 ± 4.4 | 13.7 ± 2.0 | 0.009 | 15.1 ± 3.7 | 13.2 ± 2.2 | < 0.001 | 14.0 ± 5.3 | 14.3 ± 1.6 | 0.69 |
| Heart |  |  |  |  |  |  |  |  |  |
| Mean dose (Gy) | 10.1 ± 5.3 | 9.0 ± 2.2 | 0.01 | 11.0 ± 5.6 | 10.2 ± 22.7 | 0.21 | 8.0 ± 3.9 | 7.5 ± 1.0 | 0.33 |

^a^ Vχ = the percentage of the structure volume exceeding χ Gy
